# Investigating the effect of cognitive load on the intentionality bias

**DOI:** 10.1007/s00426-024-02047-3

**Published:** 2024-11-12

**Authors:** A. E. Eisenkoeck, J. W. de Fockert, J. W. Moore

**Affiliations:** Department of Psychology, Goldsmiths, University of London, Lewisham Way, London, SE14 6NW England

## Abstract

According to Rosset’s dual-process model of intention attribution, our judgements of intentionality can be guided either by an automatic process leading to *intentional* explanations of behaviour or by a higher-level and cognitively more demanding process enabling *unintentional* explanations of behaviour. Based on this model, under conditions of compromised cognitive capacity, individuals should judge more behaviour to be intentional rather than unintentional. This prediction was tested in one lab-based experiment and one online experiment. Specifically, we investigated whether increased working memory load would lead to higher intentionality endorsement of ambiguous action when controlling for individual differences in working memory. Results of both experiments indicated no effect of working memory load on intentionality endorsement. The implications of these results for the dual-process model of intention attribution are discussed.

## Introduction

Discerning intentional from unintentional action is a key aspect of social cognition. It determines how we react to others’ behaviour and enables successful interaction between us. For example, we more readily reciprocate helping behaviour and are more likely to react aggressively toward harmful behaviour we think was done on purpose (e.g., Cushman, 2008; Gray & Wegner, 2008; Gilbert et al., 2004; Taylor et al., 1979; Swap, 1991). Our judgements of intentionality are, therefore, of considerable importance.

### Dual-process theory of intention attribution

**Fig. 1:**
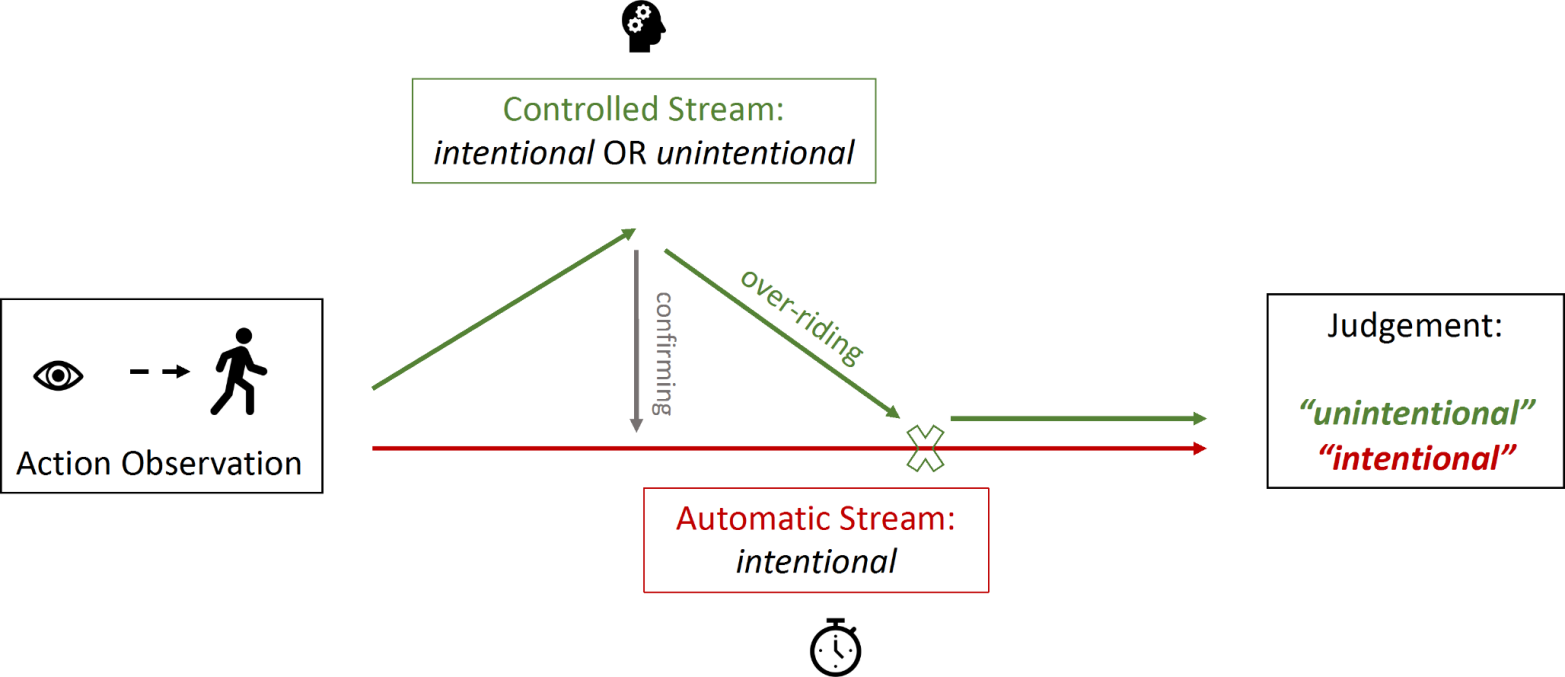
Schematic illustration of Rosset’s (2008) dual-process model of intention attribution. An automatic process leads to intentional explanations of behaviour, which can either be confirmed or inhibited and overridden by a controlled process leading to unintentional explanations of behaviour

In an attempt to capture the underlying cognitive processes of intentional reasoning, Rosset (2008) proposed a dual-process model of intention attribution. Dual-process models generally assume two types of information processing: a fast, parallel and automatic *Type 1* process and a slower, sequential and analytical *Type 2* process (Evans & Stanovich, 2013; Evans, 2003). Rosset’s (2008) dual-process model of intention attribution suggests that humans’ automatic response to others’ behaviour is to judge it to be intentional (Fig. 1). This fast, automatic (Type 1) response can be inhibited by a slower, more controlled pathway (Type 2), deploying higher-level cognitive processes. However, this can only occur when enough cognitive capacity is available and circumstances allow for the involvement of higher-level cognitive processing. Consequently, when availability of cognitive capacity is reduced or deployment of higher-level cognitive processes is otherwise prevented, more behaviour should be judged to be intentional. Indeed, some empirical data seem to confirm this prediction: when participants had to judge the intentionality of others’ behaviour under time constraints (i.e. decreased possibility to deploy higher-level processes) intentionality endorsement scores were higher than under no time constraints (Rosset, 2008). Also, in another study, intentionality endorsement scores were found to be increased when Type 2 processing was disrupted by acute alcohol intoxication (Begue et al., 2010).

Despite this apparent support for the dual-process model, there are some key limitations in these previous studies. For example, when employing time pressure it is hard to ascertain which cognitive functions are affected. It has been suggested that dealing with time pressure involves several processes, such as selective attention, affect control, and parsimony of information processing (Stiensmeier-Pelster & Schürmann, 1993). Also, previous findings suggest that when individuals have to make decisions under time pressure they experience increased anxiety (Maule et al., 2000). Therefore, a possible reason for higher intentionality endorsement scores could be due to changes in affect rather than having insufficient time to engage in controlled processing. Furthermore, an issue with alcohol manipulations is that such interventions are not well-controlled, in the sense that alcohol intoxication affects a number of cognitive functions (e.g., Field et al., 2010; Peterson et al., 1990).

Therefore, although Rosset’s (2008) results show increased intentionality endorsement under time constraints, they are inconclusive in regards to which cognitive processes are affected. The aim of the current study was to focus on and manipulate *WM load* (i.e., availability of WM capacity) specifically, and to study its role in intention attribution to ambiguous behaviour. According to Evans and Stanovich (2013), the requirement of WM is a defining feature of Type 2 processing, hence, we have reason to believe it is involved in the higher-level process of intentional reasoning.

### Dual-task design

To manipulate working memory (WM) load a dual-task approach was chosen. It is based on the assumption that available WM capacity is limited and can be flexibly distributed (see Baddeley, 1986; Miyake & Shah, 1999). When two tasks have to be completed simultaneously and both require cognitive resources, available capacity has to be split between both of them. As a result, the availability of cognitive resources for each individual task decreases compared to a single-task condition (see Brünken et al., 2002). If response patterns are contingent on available capacity then a dual-task condition should alter these. Baddeley and colleagues (Baddeley, 1986; Baddeley & Hitch, 1974; Baddeley & Logie, 1999) have employed different versions of this paradigm to empirically test their model of WM, which assumes that working memory is divided into multiple components and when two tasks rely on the same component, performance decreases.

In the current study, the role of WM in judging intentionality of ambiguous action was investigated by asking participants to complete a WM task while simultaneously being asked to judge intentionality of ambiguous action. More precisely, participants in the experimental conditions were presented with digit strings of varying lengths and were asked to retain these digits until the end of the trial, at which point participants had to indicate whether a given probe digit had been previously present (for previous studies using similar manipulation of WM load see De Fockert & Bremner, 2011; Lavie et al., 2004). The purpose of the WM manipulation was to interfere with intentional reasoning (reliant on maintaining information about an action) rather than to disrupt visual processing, which is why a digit span task (as opposed to a visual WM manipulation) was used. Also, digit span tasks require active (sub)vocal maintenance in WM and are regarded as more dependent on cognitive control mechanisms.

Whilst maintaining the digits in their memory, participants were asked to complete a version of Moore and Pope’s (2014) Ambiguous Movement Paradigm. This paradigm involves video stimuli of ambiguous finger movements, i.e. movements that can be done either intentionally or unintentionally. Participants are asked to judge the intentionality of the observed movement. The advantage of using the video stimuli is that intentional causation is not a result of a linguistic bias (induced by linguistic phrasing marking intentionality) rather than an intentionality bias, which is a has been highlited as a limitation of Rosset’s paradigm (Moore & Pope, 2014; Rosset, 2008).

To ensure individual differences in WM capacity do not confound the results, a version of Johnson et al.’s (2013) Change Localisation task to measure participants’ visual WM capacity was included. It is assumed to provide a pure measure of visual WM capacity (i.e., amount of information that individuals can retain in short-term storage) that is not heavily influenced by non-storage-specific processing strategies such as chunking or verbal rehearsal (Cowan, 2010). (Note, when talking about *WM capacity* we are referring to an individual’s WM capacity, which is assumed to be stable over time, as opposed to *available WM capacity*, which is dependent on conditions.) In the version used in the current research, a sample array of four stimuli is presented for a brief period. After a short delay, a test array is shown and participants are asked to indicate which of the four stimuli has changed colour (see Methods section for details).

### Hypothesis

If intention attribution relies on the availability of cognitive control resources, as proposed by Rosset’s (2008) dual-process model of intention attribution, then increased WM load should be associated with increased intentionality endorsement scores.

## Experiment 1

### Materials and methods

#### Participants

An a priori sample size calculation based on pilot data (f = 0.51; Power = 0.8; 3 groups) was conducted using GPower 3.0.10. It revealed a required sample size of 42 participants (12 per group). In total 46 participants took part in this lab-based experiment, but two had to be excluded because of technical issues and another two were excluded because they had indicated that they had noticed that the videos always showed the same movement. Therefore, data of 42 participants were included in the analysis (mean age in years = 20.43, SD = 4.06; 37 females). Participants were recruited through a combination of opportunity sampling and a course credit system (*n* = 35). Participants were randomly allocated to one of the three conditions. The experiment was approved by the Goldsmiths Psychology Department Research Ethics Committee.

#### Procedure and stimuli

Participants completed two tasks. All stimuli were presented on a 24-inch computer screen.

##### Change localisation task

**Fig. 2:**
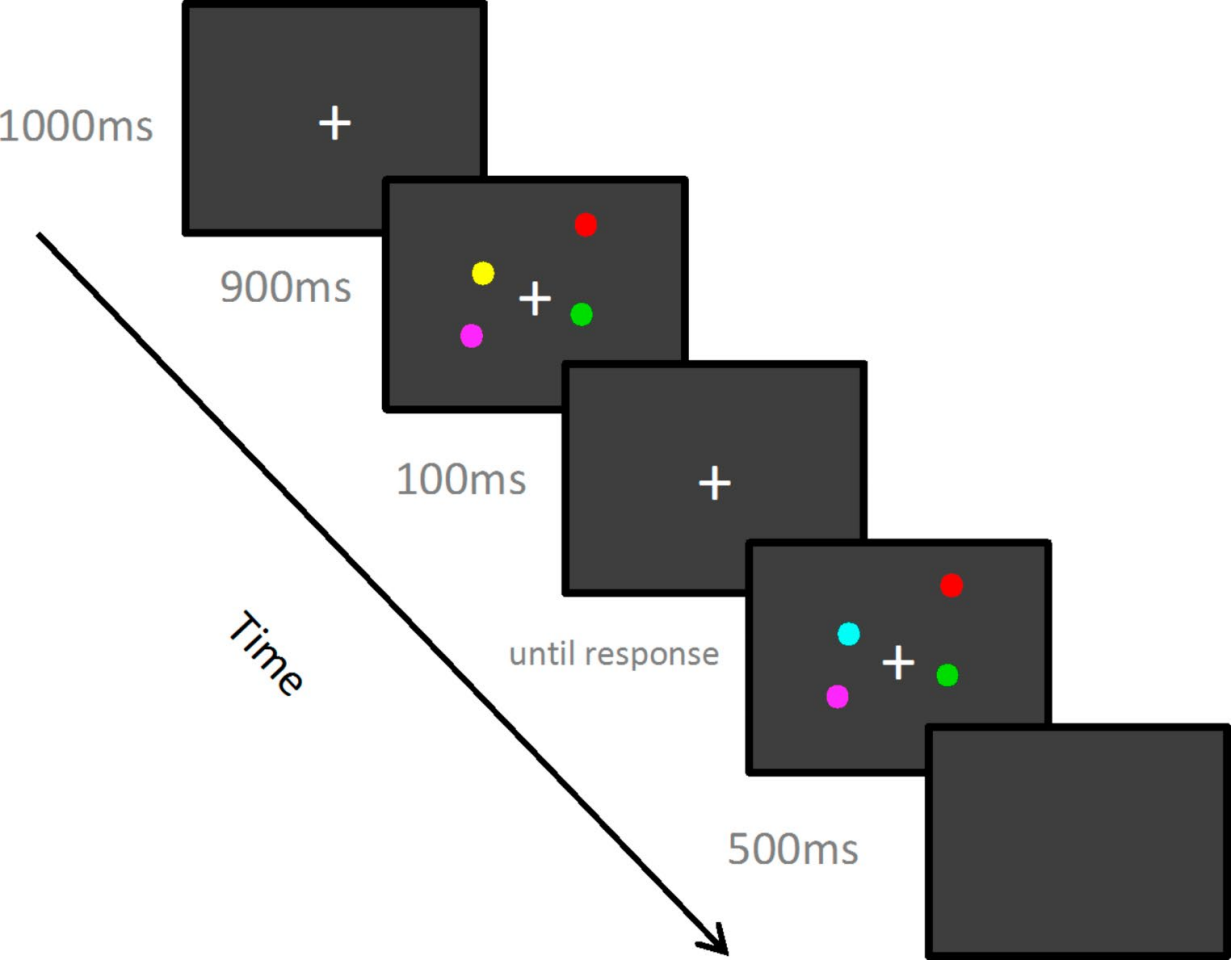
Sequence of events for one trial of the Change Localisation Task

All participants first completed a version of the Change Localisation Task (Johnson et al., 2013). The task consists of 12 practice trials and two experimental blocks of 32 trials. For each trial, participants were first presented a fixation cross for 1000 ms, subsequently they were presented with four coloured dots on random locations around the fixation cross, followed by a screen with the fixation cross only for 900 ms, and finally the fixation cross and four coloured dots on the same spatial locations as before but one of them being in a different colour. There was an inter-trial interval of 500 ms (Fig. 2; for more details on this version of the Change Localisation Task please refer to Ortells et al., 2018). Participants were asked to click on the circle they think has changed colour. For the practice trials, participants were given feedback on whether they have correctly responded to ensure they had understood the task instructions, however, no feedback was given for the experimental trials. The researcher stayed in the room for the practice trials to answer any questions but left the room thereafter.

##### Ambiguous movement paradigm

**Fig. 3:**
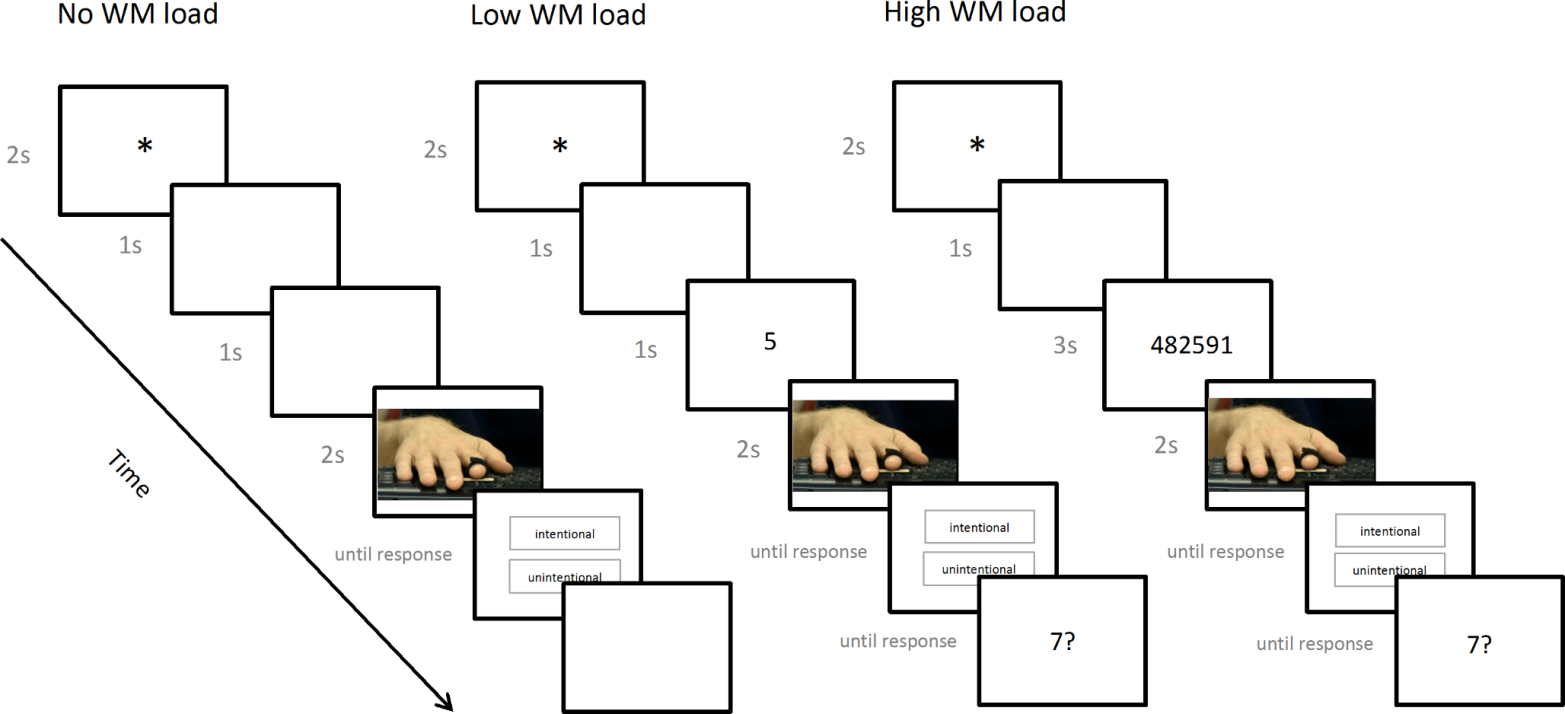
Sequence of events for one trial of each condition (NL, LL, HL) of the Ambiguous Movement Task

After the Change Localisation Task, participants were asked to complete a version of Moore and Pope’s (2014) Ambiguous Movement Paradigm either under the condition of no -, low- or high WM load. In the no WM load (NL) condition, participants were presented with a fixation cross for two seconds, followed by a blank screen for two seconds, followed by the video stimulus showing the ambiguous finger movement (three seconds), after which they had to indicate their response by saying “unintentional” or “intentional” out loud. In the load conditions, a simultaneous WM task had to be completed: Participants were shown a fixation cross for two seconds, followed either by one digit (one second; LL condition) or six digits (three seconds; HL condition) and then the video stimulus. They then had to verbally indicate first whether the movement was intentional or unintentional and then whether a single probe digit had been previously present (Fig. 3).

Before the start of the experiment, all participants were informed that the finger movement would either be intentional (person pressing the key) or unintentional (mechanism under the key pulling the finger down). In reality, the same video was shown in all trials, however, with three different movement onset delays (100ms, 400ms, 700ms) randomised across trials. It showed an unintentional movement, in which the finger was pulled down. As the same movement was shown every trial, we ensured that perceptual cues would not confound intention attribution judgements.

The researcher, who stayed in the room for this task, wrote down each participant’s responses. There were two practice trials and 24 experimental trials. After the task was completed, the participant was debriefed and thanked for their participation.

### Results

For each participant, we calculated an *intentionality endorsement score* (percentage of trials judged *intentional*) for the Ambiguous Movement Paradigm. We also calculated their *K score* for the Change Localisation Task, which was computed by dividing the hit rate by the number of trials and multiplying it by the set size of the visual displays (k = hit rate/nr of trials*set size). Consequently, each participant’s K score ranged from 0 to 4 (Table 1). Additionally, participants of the load conditions received a WM-task performance score (i.e., number of correct trials; 0–24). There were two outliers in the LL conditions, however, as they were not extreme outliers (based on inter-quartile range rule with a multiplier of 3.0), they were not excluded from analysis.

**Table 1:** Intentionality endorsement scores, K scores and WM-task performance scores for the no WM load (NL)-, low WM load (LL)- and high WM load (HL) condition with standard deviations in brackets. Possible intentionality endorsement scores range from 0 to 100, possible K scores from 0 to 4 and possible WM-task performance scores from 0 to 24

Condition	Intentionality endorsement score	K score	WM-task performance score
NL (*n* = 14)	61.61 (*10.98*)	2.87 (.*3*)	-
LL (*n* = 14)	64.88 (*15.48*)	2.71 (.*49*)	20.93 (*4.34*)
HL (*n* = 14)	61.94 (*20.90*)	2.96 (.*39*)	14.71 (*1.49*)

#### Working memory capacity

A one-way ANOVA was conducted to test whether groups differed in WM capacity. Results revealed no significant differences between groups in WM capacity (F(2, 39) = 1.42, *p* = .254, η^2^ = 0.222).

#### Manipulation check – working memory task

A non-parametric Mann-Whitney U test revealed a significant difference in the number of correct trials between the LL and the HL condition for the working memory task (U = 28, *p* < .001; Table 1). Based on this, we assume the WM load manipulation was successful.

#### Intentionality bias

In Moore and Pope’s (2014) study, participants were significantly more likely to judge ambiguous movements to be intentional than unintentional (i.e., intentionality bias). To examine whether participants in the current study showed a similar biased processing style (i.e., whether they would be significantly more likely to judge over 50% of the trials to be intentional) we conducted a one-tailed one-sample t-tests on intentionality endorsement scores with a test value of 50. Results suggested that participants judged significantly more than half of the trials to be intentional (M = 62.5 (SD = 16); t(41) = 5.065, *p* < .001); Cohen’s d = 0.782).

#### Main analysis- the effect of cognitive load on intentionality endorsement

**Fig. 4:**
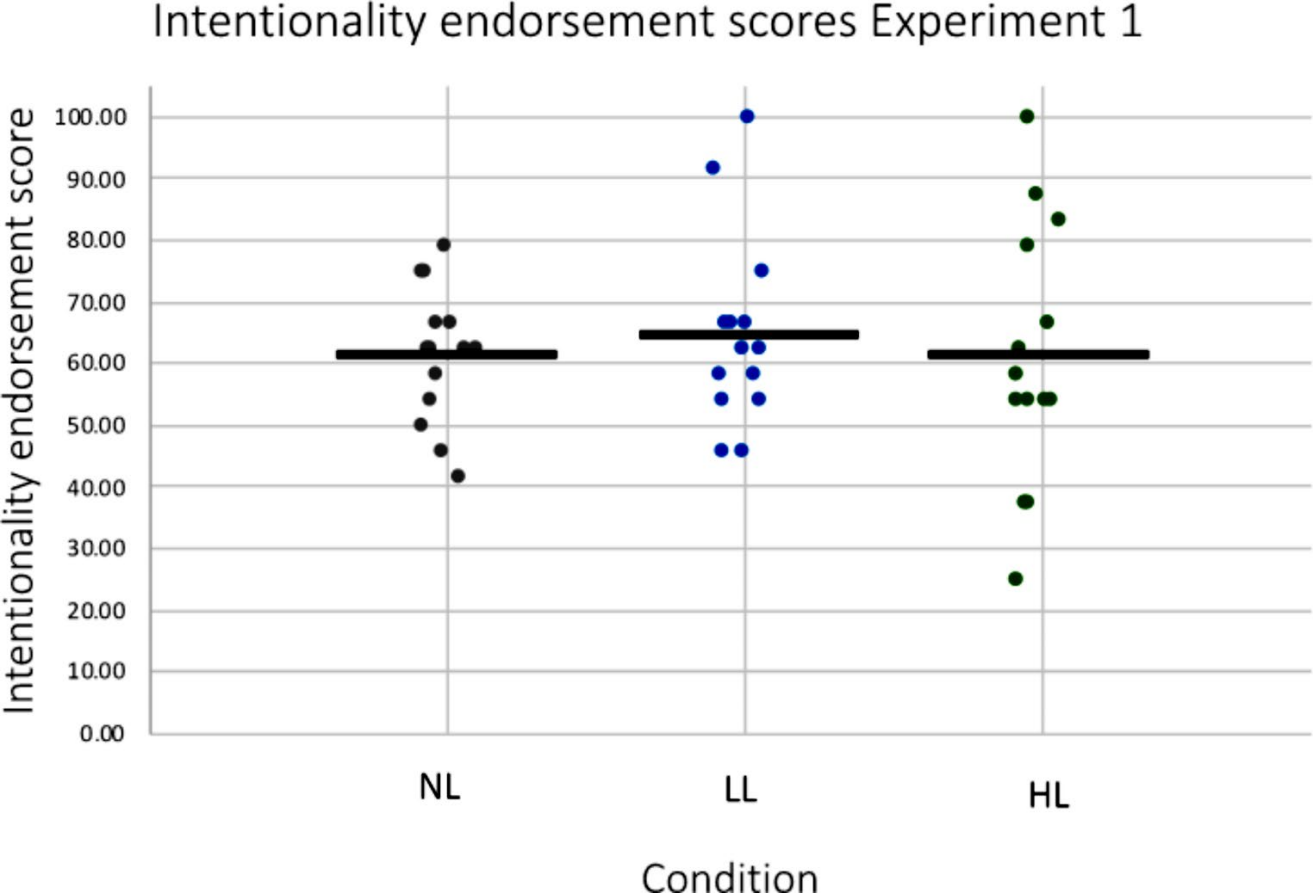
Intentionality endorsement scores for each WM load condition (no WM load, low WM load, high WM load) in *Experiment 1*. Intentionality endorsement scores reflect the percentage of trials judged to show intentional movements. The mean is indicated by a cross (x) and the median by a line

A one-way ANCOVA was conducted to determine the effect of WM load (no load, low load, high load) on intentionality endorsement scores controlling for WM capacity (K). It revealed no significant difference between groups (F(2, 38) = 0.114, *p* = .892, η^2^ = 0.006; Fig. 4). (Please note that significance of the test did not change when only performed on trials with correct responses to the WM task.)

#### Exploratory analysis: correlation working memory capacity

**Fig. 5:**
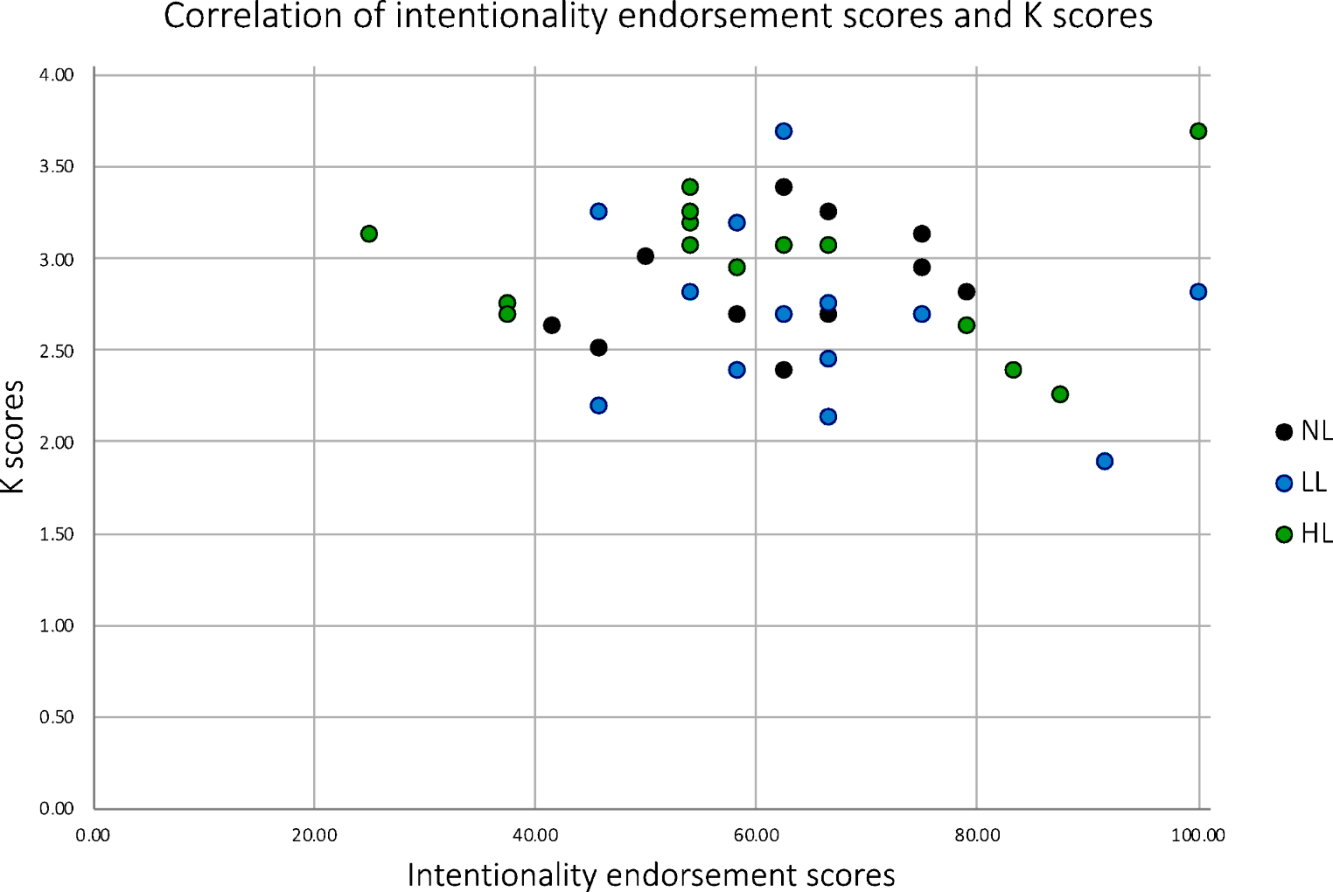
A scatterplot showing the association of K scores and intentionality endorsement scores for all three conditions for Experiment 1. K scores reflect individuals’ WM capacity (ranging from 0 to 4) and intentionality endorsement scores reflect the percentage of trials judged to show intentional movements

Involvement of WM is an essential feature of Type 2 processing (Evans & Stanovich, 2013), which is associated with making unintentional attributions. To establish whether there was a negative association between WM capacity (as a possible index for participants’ capability to engage in Type 2 processing) and intentionality endorsement, one-tailed Pearson’s correlation analyses were conducted. Analyses were conducted for each condition separately (*n* = 14) as well as pooled across groups (*n* = 42) in order to increase the sample size. Results suggested no association between working memory capacity and intentionality endorsement for each group separately (NL: *r* = .275, *p* = .171; LL: *r*=-.293, *p* = .155; HL: *r*=-.099, *p* = .369) nor for all three groups combined (*r*=-.131, *p* = .204; Fig. 5).

### Preliminary discussion

In *Experiment 1* we investigated the effect of increased WM load on intentionality endorsements for ambiguous action. It was predicted that WM load would lead to increased intentionality endorsement scores. In a between-participants design with three groups that did not differ in terms of WM capacity, we compared intentionality endorsement scores under conditions of no WM load, low WM load and high WM load. All groups showed a bias towards judging the movement to be intentional. Results of the effect of WM load on intentionality judgements are not in line with our predictions. It is possible that the parameters used for the a priori sample size calculations were inaccurate and, hence, our sample size was too low. Therefore, in *Experiment 2*, we decided to run a second experiment to test our hypothesis.

Our sample size calculations were based on detection of a correlation between WM capacity and intentionality endorsement. As argued by Evans and Stanovich (2013) the involvement of WM is essential for Type 2 processing. According to Rosset’s dual-process unintentional explanations for behaviour are based on Type 2 processing. In light of this, we had formed a second hypothesis: Individuals with higher WM capacity (i.e. individuals who find easier to engage in Type 2 processing) will show overall lower intentionality endorsement scores. Results from *Experiment 1* do not show a significant correlation, although this could be due to the small sample size (*N* = 42), in light of which we decided to investigate the association in a larger sample.

## Experiment 2

### Materials and methods

#### Participants

Based on the results from *Experiment 1*, the sample size required to detect a significant negative correlation between WM capacity (*K*) and intentionality endorsement scores pooled across all three conditions (for simplicity and feasibility) was calculated using GPower 3.0.10 (*r* = .131, Power = 0.8; one-tailed hypothesis), which resulted in a required sample of 358 participants. Participants were recruited via *Testable Minds*, an online platform on which participants get reimbursed monetarily for their participation. The study was online for 20 days during which a sample size of 329 participants (mean age in years: M = 34.96; SD = 11.83; 143 female) was reached, which is slightly below the a priori calculated sample size. Participants were randomly allocated to one of three conditions: no load condition (*n* = 107), low load condition (*n* = 108), or high load condition (*n* = 114). The study was approved by the Goldsmiths Psychology Department Research Ethics Committee.

#### Procedure and stimuli

*Experiment 2* was an online replication of *Experiment 1*, i.e. online versions of the same tasks were conducted: After reading the online information sheet and consent form, participants completed an online version of the Change Localisation Task. Thereafter, they were asked to complete the Ambiguous Movement Paradigm under no WM load (NL), low WM load (LL) or high WM load (HL).

For details of both paradigms, please refer to *Experiment 1*. As this was an online experiment and, hence, screen size could not be controlled, an average screen size and distance from the screen was estimated. Based on this estimate, a window with a fixed size (pixels) was created on which stimuli were displayed. This ensured that, for the Change Localisation Task, the angles of the circle-positionings relative to the fixation cross would not differ greatly between participants. Because of a technical error only one of two experimental blocks of the Change Localisation was presented, i.e. the number of trials was 32 in total. (As can be seen from the Results section, performance on this shorter version of the task was similar to the full task run in *Experiment 1*.)

### Results

As in *Experiment 1*, for each participant, a *K score* (WM capacity) and an *intentionality endorsement score* (percentage of trials judged *intentional*) were calculated. Participants from the two WM load conditions additionally received a *WM-task performance score*. Six extreme outliers who had significantly poorer WM-task performance scores than the other participants in their group (based on inter-quartile range rule multiplier of 3.0), were excluded from the analysis, as such scores could be a sign of inattentiveness or misunderstanding of the task instructions. All of them were from the LL group. Excluding them resulted in a new sample of 323 participants.

**Table 2:** Intentionality endorsement scores, K scores and WM-task performance scores for experiment 2 for the no WM load (NL)-, low WM load (LL)- and high WM load (HL) condition with standard deviations in brackets. Possible intentionality endorsement scores range from 0 to 100, possible K scores from 0 to 4 and possible WM-task performance scores from 0 to 24

Condition	Intentionality endorsement score	K score	WM-task performance score
NL (*n* = 107)	59.07 (*17.23*)	2.79 (*0.73*)	-
LL (*n* = 102)	60.74 (*20.96*)	2.94 (*0.52*)	22.03 (*3.19*)
HL (*n* = 114)	63.38 (*20.75*)	2.88 (*0.55*)	*21.41 (3.15*)

#### Working memory capacity

A one-way ANOVA was conducted to test whether groups differed in WM capacity. Results revealed no significant differences between groups in WM capacity (F(2, 320) = 1.57, *p* = .209; η^2^ = 0.10).

#### Manipulation check – working memory task

Participants in the LL- and the HL condition responded correctly to a large proportion of trials of the WM task, with the LL group scoring slightly higher (Table 2). A one-tailed Mann-Whitney U test revealed that this difference was statistically significant (U = 4639.5, *p* = .008). Based on this, we assume that the WM load manipulation was successful.

#### Intentionality bias

To examine whether participants in this experiment showed a bias in their intentionality judgements (i.e., whether they would be significantly more likely to judge over 50% of the trials to be intentional) we conducted a one-tailed one-sample t-tests on intentionality endorsement scores with a test value of 50. Results suggested that participants judged significantly more than half of the trials to be intentional (M = 61.12 (SD = 19.75); t(322) = 10.12, *p* < .001; Cohen’s d = 0.563).

#### Main analysis- the effect of cognitive load on intentionality endorsement

**Fig. 6:**
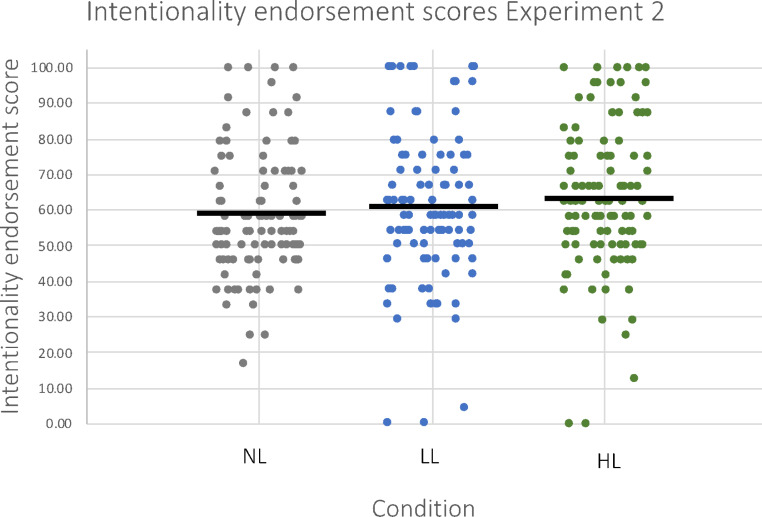
Intentionality endorsement scores for each WM load condition (no WM load, low WM load, high WM load) in *Experiment 2*. Intentionality endorsement scores reflect the percentage of trials judged to show intentional movements. The mean is indicated by a cross (x) and the median by a line

A one-way ANCOVA was conducted to determine the effect of WM load (no WM load, low WM load, high WM load) on intentionality endorsement scores controlling for working memory capacity (K). Although the trend pointed in the right direction, our analysis revealed no significant difference between groups (F(2) = 1.49, *p* = .227; η^2^ = 0.009; Fig. 6).

#### Correlation WM capacity and intentionality endorsement

**Fig. 7:**
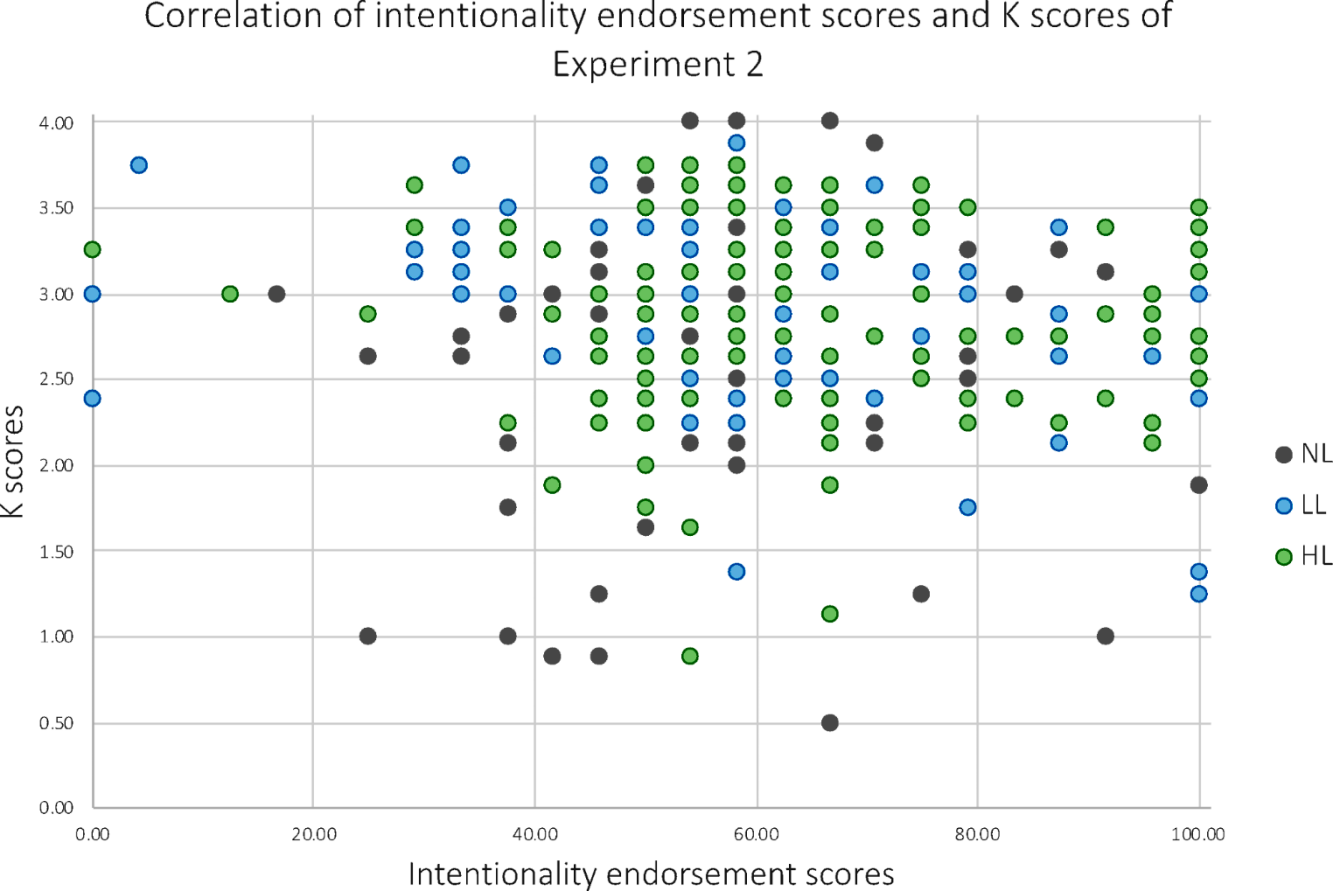
A scatterplot showing the association of K scores and intentionality endorsement scores for Experiment 2. K scores reflect individuals’ WM capacity (ranging from 0 to 4) and intentionality endorsement scores reflect the percentage of trials judged to show intentional movements

To investigate the relation between WM capacity (*K*) and intentionality endorsement, a one-tailed Pearson’s correlational analysis was conducted. It revealed no significant correlation between K scores and intentionality endorsement scores, however, results indicated a trend in the predicted direction (*r*=-.088, *p* = .057; Fig. 7).

#### Exploratory analysis: correlation WM capacity and intentionality endorsement for each condition separately

As individual differences in WM capacity might play a role only under certain conditions (e.g., under NL when participants can make full use of their WM capacity, *or* under conditions of WM load, as only then individual differences in WM become apparent), in this part of the analysis we looked at the relation between WM capacity and intentionality endorsement in each group separately (one-tailed).

##### i) No load condition

**Fig. 8:**
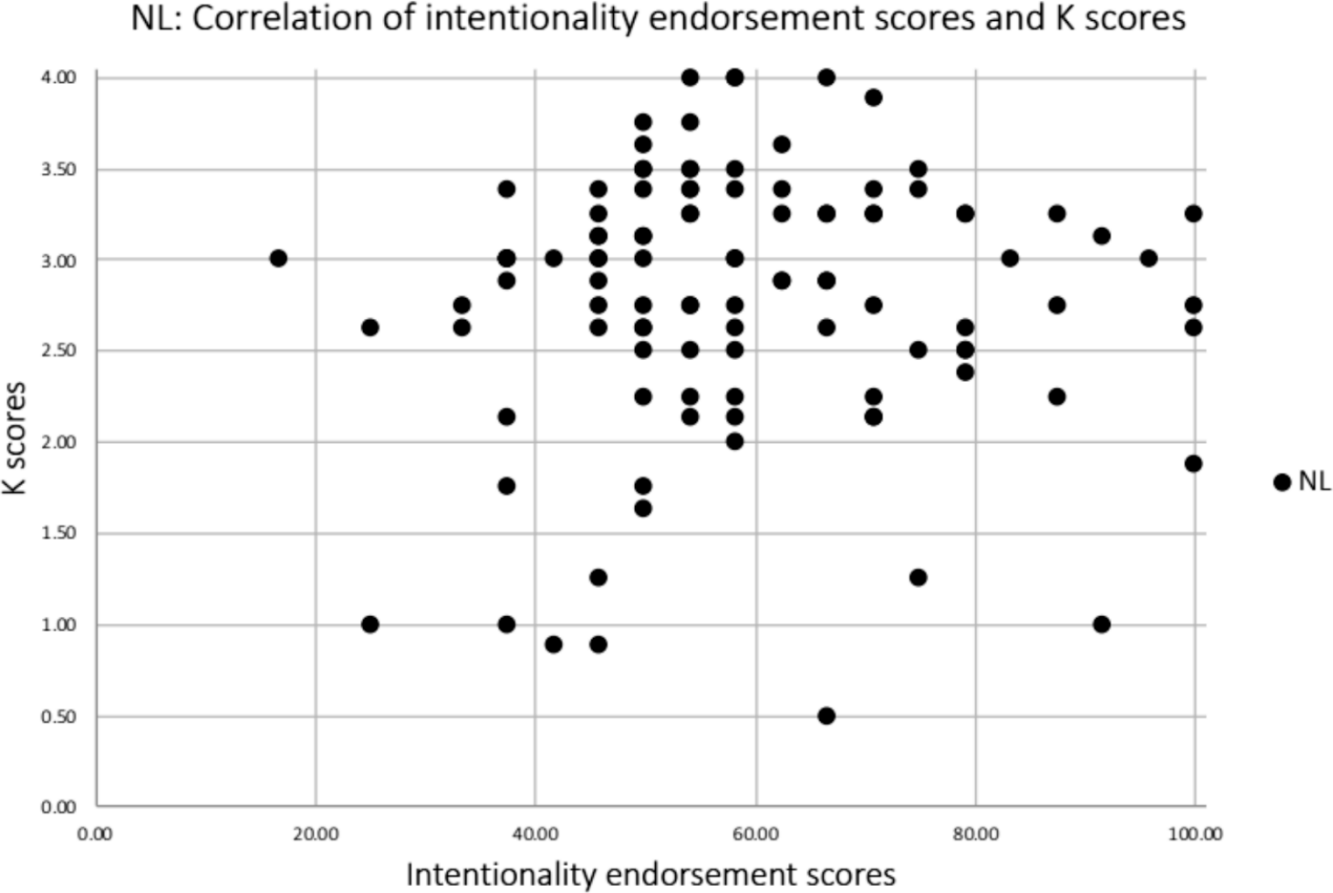
A scatterplot showing the association of K scores and intentionality endorsement scores in Experiment 2 for the no WM load condition only. K scores reflect individuals’ WM capacity (ranging from 0 to 4) and intentionality endorsement scores reflect the percentage of trials judged to show intentional movements

There was no significant correlation between intentionality endorsement scores and WM capacity (K) in the NL condition (*r* = .046, *p* = .319; Fig. 8).

##### ii) Low load condition

**Fig. 9:**
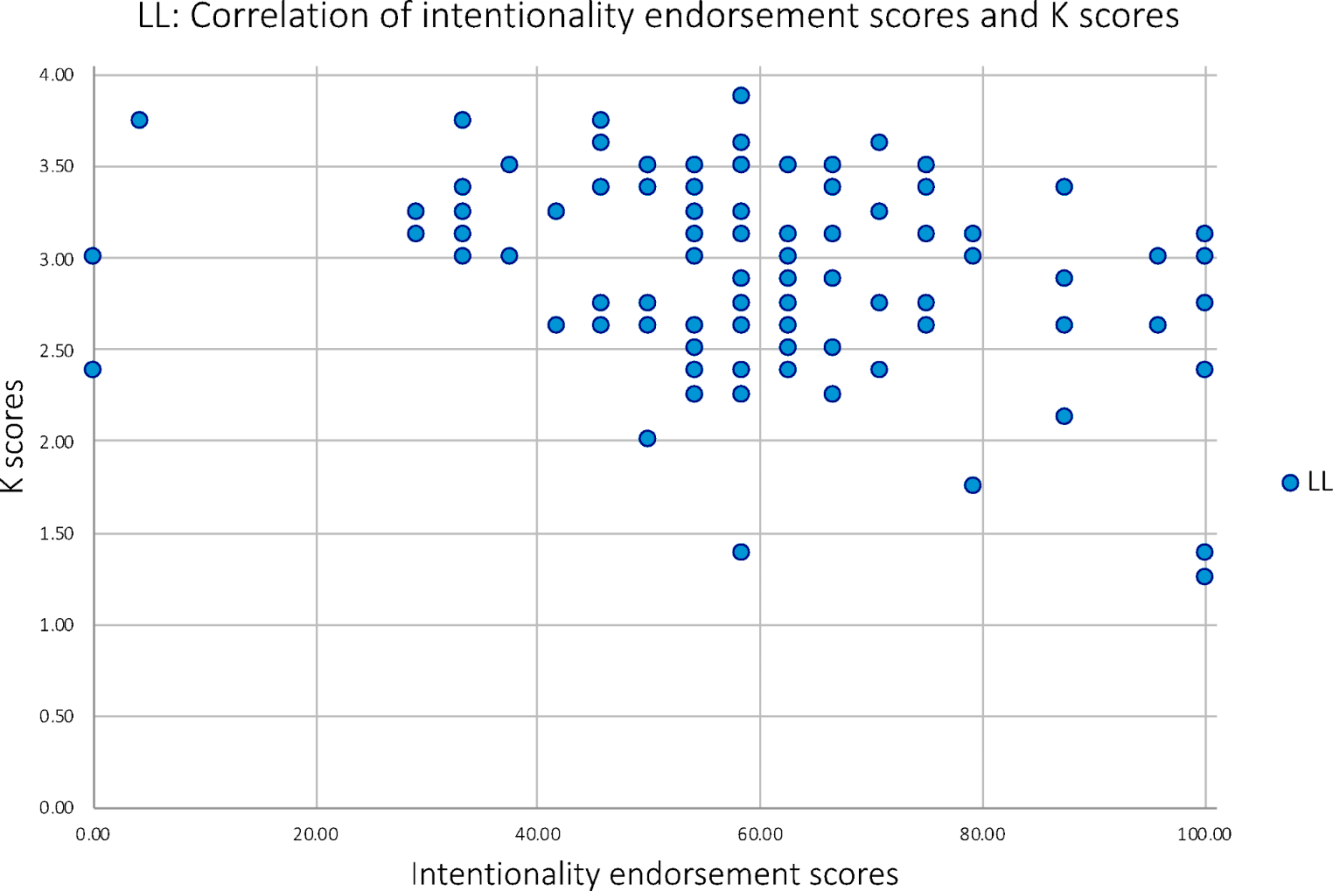
A scatterplot showing the association of K scores and intentionality endorsement scores in Experiment 2 for the low WM condition only. K scores reflect individuals’ WM capacity (ranging from 0 to 4) and intentionality endorsement scores reflect the percentage of trials judged to show intentional movements

There was a significant negative correlation between intentionality endorsement scores and WM capacity (K) in the LL condition (*r*=-.291, *p* < .001; Fig. 9). (This effect was not dependent on the exclusion of outliers.)

##### iii) High load condition

**Fig. 10:**
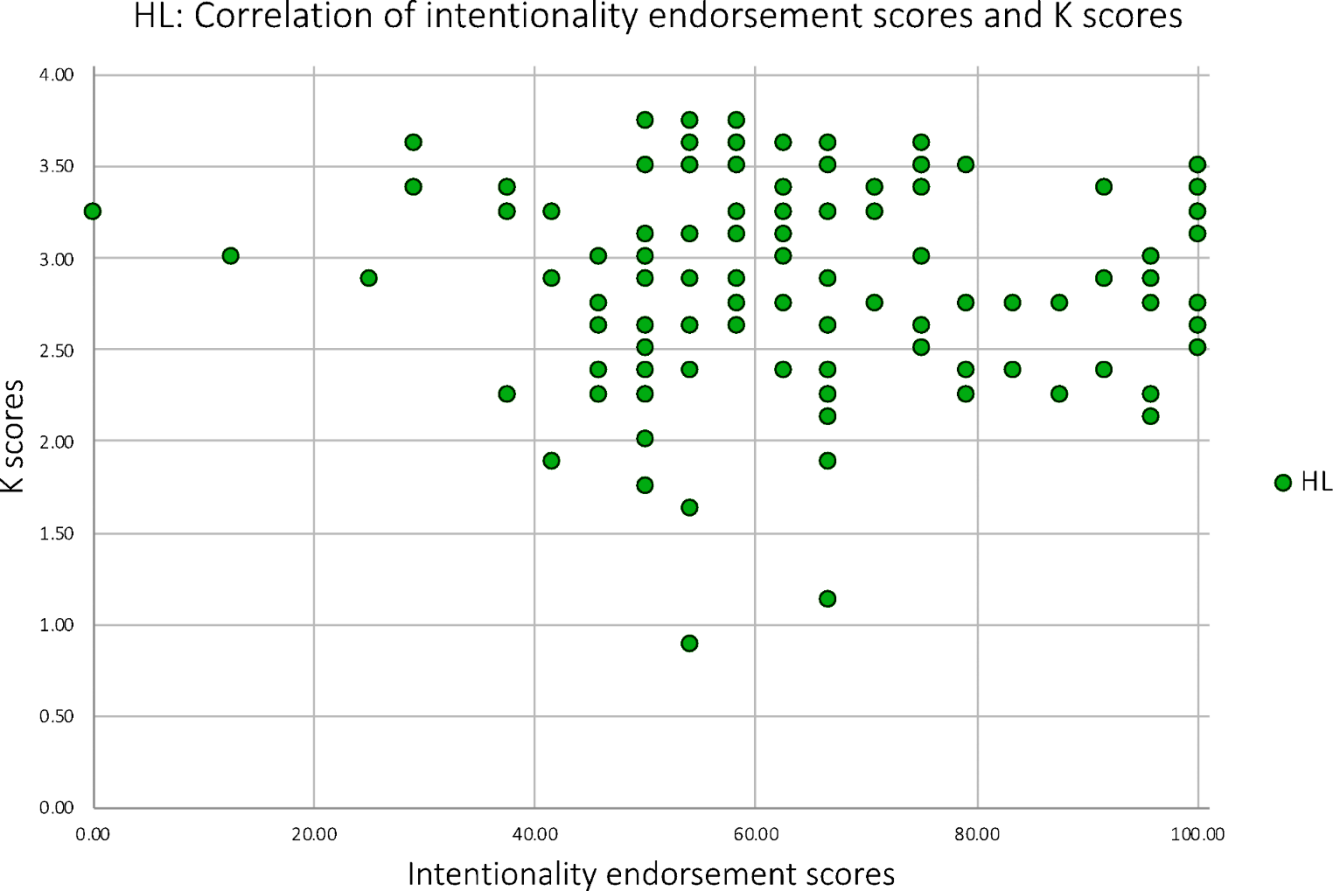
A scatterplot showing the association of K scores and intentionality endorsement scores in Experiment 2 for the high WM load condition only. K scores reflect individuals’ WM capacity (ranging from 0 to 4) and intentionality endorsement scores reflect the percentage of trials judged to show intentional movements

There was no significant correlation between intentionality endorsement scores and WM capacity (K) in the HL condition (*r*=-.078, *p* = .201; Fig. 10).

### Preliminary discussion

In *Experiment 2* we set out to re-test our hypothesis from *Experiment 1*. All groups showed a bias towards judging the movement to be intentional. Although our results of the between-group analysis go in the predicted directions and intentionality endorsement scores are higher under conditions of increased WM load, results are not significant.

In addition, we tested whether WM capacity was negatively correlated with intentionality endorsement. Whereas there is no significant correlation when pooled across groups, an exploratory analysis revealed a significant negative correlation between WM capacity and intentionality endorsement scores in the LL condition only. One possible explanation is that only under a condition in which WM capacity is compromised (i.e., an individual’s entire WM capacity cannot be dedicated to the task) but not compromised enough to demand most WM capacity of all participants including high WM-capacity individuals, individual differences in WM capacity play the biggest role.

In addition, on average, participants in the HL condition of *Experiment 2* scored relatively high on the WM task (compared to *Experiment 1* and previous results), which suggests that these participants dedicated a large part of their WM capacity to the WM-task. This alludes to the possible role of thinking disposition (i.e., tendency for actively open-minded thinking/ need for cognition or lack thereof; West, Toplak, & Stanovich, 2008), as it influences which task the available cognitive are allocated to. However, it has to be emphasised here that at this stage such possibilities remain speculative, as we did not specifically test for the involvement of thinking disposition.

## Discussion

Rosset (2008) proposed a dual-process model for intention attribution which suggests that when observing an ambiguous action, humans automatically attribute intent. This attribution can, however, be inhibited and over-ridden by a higher-level process, given enough cognitive resources are available. A prediction from this model is that decreasing the availability of such cognitive resources would lead to increased intentionality endorsement. Rosset gave no clear indication as of which cognitive resources were likely to be involved in judging intentionality of ambiguous action, however, according to Evans and Stanovich (2013) a defining feature of Type 2 processing is the dependency on WM. Therefore, in two experiments, the role of WM load on judging intentionality of ambiguous action was investigated. In line with Moore and Pope’s (2014) results, in both experiments, participants of all three conditions were more likely to judge the ambiguous finger movements to be intentional than unintentional, which suggests an automatic tendency to perceive ambiguous behaviour to be intentional. However, the null hypothesis was not rejected: Participants did not show higher intentionality endorsement under conditions of increased WM load.

Apart from potential design-limitations (discussed below), we identify two explanations for the lack of effect of WM load on intentionality endorsement:1) Rosset’s dual-process model is incomplete or inaccurate, or 2) individual differences such as thinking disposition play a bigger role in judging intentionality of ambiguous action and, hence, “over-shadow” any relation between WM and intentionality endorsement.

### Dual-process model - too simplistic a model?

A possible explanation for why manipulating WM capacity did not have an effect on intentionality endorsement could be that Rosset’s (2008) dual-process model is a too simplistic or is even an incomplete model of intention attribution. The model implies two basic predictions: (1) An unintentional response requires the involvement of Type 2 processing, and (2) the default heuristic response is intentional.

We suggest that the current dual-process model of intentional attribution could benefit from refinement. A more nuanced version of the model might be more open to both intentional and unintentional heuristic judgments, acknowledging that our default responses are influenced by contextual factors and experience. While some actions may indeed evoke a default intentional response, this may be more appropriately viewed as something that can develop and evolve with knowledge and exposure to different situations.

Alternatively, it could be considered whether intention attribution is rather better accommodated by a single-system framework. The main line of argument here is that the dichotomy proposed by dual-process theories does not reflect the variety of processes of human reasoning but that the two processes might be unified within a single-system (Osman, 2004). Single-system accounts, as for example connectionist models or further developments of it could maybe better explain intentional reasoning based on the idea that individuals have cognitive representations (i.e., connections) of the intentionality of others’ actions (e.g., Cleeremans & Jiménez, 2002). The quality of such representations is dynamic, being altered by experience and learning. In contrast to default and analytical processing of behaviour, such an approach would suggest that differences in reasoning are explained by differences in the quality of representations, in other words, how easily accessible the information is.

### Other individual differences: thinking disposition

Furthermore, as already mentioned above, it is possible that other individual differences, such as for example thinking disposition “over-shadow” the role of availability of WM capacity in judging intentionality of ambiguous action. For example, the participants’ ‘felt need’ to override an automatic response (i.e., detection of possible violation of normative correct response) might have differed between groups. Perhaps, although participants in the NL condition had capacity available to detect a heuristic response and to give an analytical one instead, they might have not felt the need to do so (Stanovich & West, 1997, 1998, 2008). Similarly, groups might have differed in their preferred target for the allocation of cognitive resources. Our exploratory analysis in *Experiment 2* revealed a significant correlation between individuals’ WM capacity and intentionality endorsement scores in the LL condition only. This could potentially suggest that only participants of this condition dedicated a large enough proportion of their WM capacity towards the intentionality judgement task for individual differences in WM capacity to make a difference. Furthermore, the difference between WM-task performance scores of participants of the LL- and HL condition was not as big as in *Experiment 1* and previous experiments, which could indicate that in *Experiment 2* participants of the HL condition dedicated a large part of their WM capacity to the WM-task.

### Limitations and future directions

One limitation of the current research is that there was no measure in place to test whether participants had noticed that all videos showed the same movement as expliclitly asking them could have primed an answer. If participants in the lab-based experiment spontaneously reported that videos were the same, they were excluded from the study, which amounted to 4.35% of the sample. In the online experiment, such participants would not have been picked up, hence, there is the possiblility that some participants who noticed that videos were the same were included in the analysis. However, we do not feel this is a problem as noticing that the videos showed the same movement was not reliant on WM condition, i.e., the affected participants are likely spread across groups.

Another limitation, specific to *Experiment 2*, is that it was an online experiment and therefore, it was impossible to control participants’ environment. Judging from the high mean WM-task performance scores and K scores, we assume participants paid attention to the tasks. However, we cannot know whether they used additional aids such as taking notes for the WM-task.

There may also be limitations of the finger judgement task, in particular, concerning the nature of the stimuli we used. The stimuli focused in on a finger movement (see Fig. 3). This was selected so as to minimise contextual cues or social biases. However, in doing so, it is possible that the stimuli lack the agentic properties central to Rosset’s original hypothesis. As such, the absence of clear cues indicating intentional agency may have reduced the likelihood of triggering the intentionality bias. Future research could incorporate alternative designs, such as presenting images of agents performing actions or using storyboard representations, to strengthen the sense of agency and more robustly test for intentionality attributions.

It is worth noting that judgements of intentionality are primarily judgements of a *social* nature – the behaviour of other agents is being assessed. And yet, in our experiments our load manipulation was non-social. It is possible that the lack of an effect of our load manipulation was due to the manipulation taxing the wrong kind of cognitive process. As such, it would be fruitful for future research to use a secondary *social* load manipulation, which might provide a stronger (and more appropriate) test of the dual-process model of intention attribution.

## Conclusion

In two experiments we investigated the effect of WM load on intentionality endorsement of ambiguous action. In neither of the experiments, WM had an effect on intentionality endorsement. This undermines Rosset’s (2008) dual-process model of intention attribution, which we argue is incomplete and needs to be revised.

## Data Availability

Research data supporting the results of this manuscript can be found here: https://osf.io/jtgke/
